# A Novel Angiotensin I-Converting Enzyme Inhibitory Peptide Derived From Goat Milk Casein Hydrolysate Modulates Angiotensin II-Stimulated Effects on Vascular Smooth Muscle Cells

**DOI:** 10.3389/fnut.2022.878768

**Published:** 2022-04-11

**Authors:** Zijiao Qiao, Jiaqi Wang, Zeqi He, Lina Pan, Konglong Feng, Xiaoyu Peng, Qianru Lin, Yu Gao, Mingyue Song, Sufang Cao, Yunjiao Chen, Yong Cao, Guo Liu

**Affiliations:** ^1^Guangdong Provincial Key Laboratory of Nutraceuticals and Functional Foods, College of Food Sciences, South China Agricultural University, Guangzhou, China; ^2^Ausnutria Dairy (China) Co., Ltd., Changsha, China; ^3^College of Horticulture, South China Agricultural University, Guangzhou, China

**Keywords:** goat milk casein hydrolysates, Angiotensin I-converting enzyme inhibitory peptide, amino acid sequence, hypertensive, vascular smooth muscle cells, Angiotensin II

## Abstract

Hypertension is a major risk factor leading to cardiovascular disease, and is frequently treated with angiotensin I-converting enzyme (ACE) inhibitory peptides. The objective of this study was to separate and identify an ACE-inhibitory peptide from goat milk casein hydrolysates, and to evaluate its potential for improving angiotensin II (Ang II)-mediated adverse effects on vascular smooth muscle cells (VSMCs). A novel ACE-inhibitory peptide with the highest activity from the goat milk casein hydrolysates as determined by four steps of RP-HPLC was purified and identified as Phe-Pro-Gln-Tyr-Leu-Gln-Tyr-Pro-Tyr (FPQYLQYPY). The results of inhibitory kinetics studies indicated that the peptide was a non-competitive inhibitor against ACE. Gastrointestinal digest *in vitro* analysis showed that the hydrolysate of FPQYLQYPY was still active after digestion with gastrointestinal proteases. Moreover, we found that the peptide could significantly inhibit the proliferation and migration of Ang II-stimulated VSMCs. Further transcriptomic analysis revealed that differentially expressed genes (DEGs) were enriched in the cardiovascular disease-related pathways, and that the peptide may have the ability to regulate vascular remodeling. Our findings indicate the potential anti-hypertensive effects of FPQYLQYPY, as well-implicate its role in regulating vascular dysfunction.

## Introduction

Hypertension is a major preventable risk factor for cardiovascular disease and all-cause mortality globally. The prevalence of hypertension is increasing worldwide due to unhealthy diet and lack of exercise (1), and in China, hypertension was highly prevalent but remained undertreated and uncontrolled (2). Serious health implications coupled with pervasiveness, make the prevention and treatment of high blood pressure is an onus to society.

Angiotensin I-Converting enzyme (ACE) is a key enzyme in the regulation of blood pressure and is the subject of most research (3). It can cleave Angiotensin II (Ang II) to form Angiotensin II (Ang II) in the renin-angiotensin system (RAS) pathway (4, 5). Ang II is a key active vasoconstrictor component of RAS and is harmful to vascular health (6). Vascular smooth muscle cells (VSMCs) are an important cellular component of the blood vessel wall that not only protect the structure of blood vessels, but also play a role in regulating the diameter and blood flow of the blood vessel. Ang II overstimulates VSMCs, resulting in abnormal cell proliferation, migration, oxidative stress, and inflammation (6). These processes promote vascular remodeling, leading to hypertension and atherosclerosis (7). Therefore, vascular smooth muscle cells are used as a cell model to evaluate the mechanism of antihypertensive peptides, and angiotensin II is used to construct the cell model *in vitro* (8).

The current standard care for the treatment of hypertension includes drugs which are mostly synthetic ACE-inhibitors, such as captopril and enalapril, to name a few. But, regardless of their anti-hypertensive effects, synthetic ACE-inhibitors are accompanied by undesirable side effects, including headache, cough, dizziness, fatigue, and diarrhea (9). Therefore, patients are encouraged to consider the use of bioactive peptides from functional foods to avoid side-effects.

It has been shown that bioactive peptides from milk proteins have potential health benefits for humans and are used in health-promoting food as well as drug applications (10). Based on their activity, these peptides can be divided into categories based on their properties, such as ACE-inhibition, anti-oxidation, antibacterial, mineral-binding, opioid, and immunomodulation, etc. (11). Studies have shown that peptides with antihypertensive ability may exert antihypertensive effects through a variety of mechanisms (5), and amongst the polypeptides with antihypertensive effects that have been confirmed, ACE-inhibition is the main mechanism. In recent decades, biologically active peptides with ACE inhibitory effects have been extensively studied.

Milk protein is one of the good sources of ACE-inhibitory peptides, and various ACE-inhibitory peptides have been purified and identified from it using enzymatic hydrolysis or fermentation (12). The beneficial effects of ACE-inhibitory peptides on hypertension are not limited to ACE inhibition, but also can improve vascular function disorder (13). So far, research on milk-derived bioactive peptides has focused mainly on bovine milk protein and products (14). To the best of our knowledge, the bioactive peptides isolated from goat milk casein hydrolysates have not been studied in depth, especially the ACE-inhibitory peptide (15).

Research on the separation and *in vitro* activity of ACE-inhibitory peptides in goat milk remains insufficient (16–18), and the need for more research is obvious.

In this study, we separated the ACE-inhibitory peptide from goat milk casein hydrolysates using RP-HPLC and identified the amino acid sequence of a novel peptide using MALDI TOF-MSMS and Protein Sequencer. We evaluated the ability of the purified peptide to improve the Ang II-mediated adverse effects on VSMCs and assessed its potential to improve vascular dysfunction.

## Materials and Methods

### Materials

Goat micellar casein (gMCC60) was provided by Ausnutria (Netherlands) and contained 63% protein and 22% lactose.

Trypsin (10–20% activity protein) was purchased from Novozymes (Bagsvaerd, Denmark). Pepsin (10000 NFU mg^−1^) was purchased from China Pangbo Biological Engineering Co., Ltd. (Nanning, China). Angiotensin Converting Enzyme from rabbit lung, hippuryl-L-histidyl-L-leucine (HHL), and α-amylase (109.8 U mg^−1^) were purchased from Sigma-Aldrich (USA). The Dulbecco's modified Eagle's medium (DMEM), phosphate-buffered saline (PBS), 0.25% trypsin-EDTA, penicillin-streptomycin solution, and Fetal bovine serum (FBS) were purchased from Gibco Life Technologies (Grand Island, NY, USA). Angiotensin II (Ang II) and the Cell Counting Kit-8 were purchased from MedChemExpress (MEC, NJ, USA).

### Preparation of Goat Milk Casein Hydrolysates

The hydrolysis process parameters were according to the reported method with some modifications (12). The specific process was to first dissolve the goat milk casein in distilled water until its concentration was 10%. When the solution was heated to 40°C, the pH was adjusted to 8.0 by adding 6 M NaOH. Then, trypsin was added to the reaction mixture at the ratio of 0.4% (enzyme: substrate, w/w, protein basis). The pH of the reaction mixture was maintained by continuous addition of 6 M NaOH during enzymatic hydrolysis. After enzymolysis for 2.5 h, the enzyme was inactivated by heating for 30 min at 90°C, and the hydrolysates were then freeze-dried and stored at −20°C.

### ACE-Inhibitory Activity Assay

ACE inhibitory activity was measured using an RP-HPLC method with Hippuryl-His-Leu (HHL) as a substrate, which generated hippuric acid (HA) under enzymatic hydrolysis (19). The sample was dissolved in a borax-boric acid buffer containing 0.15 M NaCl (pH = 8.3). Firstly, add 10 μL ACE (0.2 U) to the sample tube and blank tube, respectively. At the same time, add 10 μL of sample to the sample tube, and add 10 μL of buffer and 80 μL of 1 M HCl to the blank tube, then pre-incubate at 37°C for 5 min. The reaction was initiated by adding 30 μL HHL (6.5 mM) and incubated for 60 min at the same temperature until 80 μL 1 M HCl was added to terminate the reaction.

ACE inhibitory activity (%) was confirmed by monitoring the formation of HA at 228 nm as detected by RP-HPLC on a C18 column (250 × 4.6 mm, 5 μm, Ecosil). The mobile phase was 25% acetonitrile and 75% water with 0.01% trifluoroacetic acid with a flow rate of 1 mL/min. The inhibition activity was calculated using the following equation:


$$Inhibition\;activity\left(\%\right)=\frac{A-B-A_{0}}{A-A_{0}}\times 100\%$$

Where, A = the HA peak area of the control; B = the HA peak area of the sample; A_0_ = the HA peak area of control with HCL before pre-incubated.

The inhibitory activity of the hydrolysates or collected fractions was expressed as percentage of ACE inhibition at a given protein concentration (20).

### Separation and Purification of ACE-Inhibitory Peptides

The hydrolysate was subjected to a four-step separation process. First, the hydrolysate was separated by a reverse-phase (RP) C18 column (20 × 450 mm, 10 μm, Macherey Nagel, France). Elution was performed with mobile phase A (0.1% trifluoroacetic acid in water) and mobile phase B (0.1% trifluoroacetic acid in acetonitrile) with a gradient of 10–48% B at a rate of 10 mL min^−1^ for 66 min, and 90% B in the next 5 min. Second, the active fraction was further purified by a RP Shim-pack PRC-ODS(K) column (30 × 250 mm, 15 μm, Shimadzu), and the elution was performed with 25–60% solvent B for 70 min. Third, the active fraction peptide was further purified by a RP ECOSIL C18 column (300 × 20 mm,10 μm, Germany), and the elution was performed with 30–43% solvent B for 60 min. Last, the fraction solution with the highest ACE-inhibitory activity was further separated using an analytical HPLC equipped with a C18 column (5 μm, 4.6 × 250 mm, Ecosil), and the elution was performed with 25 to 30% solvent B for 60 min. The peak of highest activity was further analyzed using an analytical RP-HPLC equipped with the same C18 column, and the elution was performed with 10–60% solvent B for 40 min, 90% B for the next 10 min, and 10% B in the last 10 min.

The absorption peaks of every step were fractionated, collected and lyophilized in order to measure the ACE-inhibitory activity. The eluted samples were monitored at a dual wavelength of 214 and 280 nm.

### Identification of the ACE Inhibitory Peptides

The molecular mass of the peptide with the highest ACE-inhibitory activity was determined using an ABI 4800 MALDI-TOF-MS/MS (Bruker Daltonik GmbH, Bremen, Germany). The N-terminal sequence of the purified peptides was identified by Protein Sequencer using the Edman degradation procedure (PPSQ-53A, Shimadzu, Japan).

### Peptide Syntheses

The peptide (FPQYLQYPY) was synthesized by Synpeptide Co., Ltd (Nanjing, China). The purity of the synthesized peptide was 98%, as evaluated by HPLC and the molecular mass was determined by LC/ESI–MS.

### Inhibitory Kinetics Study

To clarify the inhibitory mechanism of the most potent purified peptide on ACE, different concentrations of the ACE inhibitory peptide were added to each reaction mixture, according to a previously reported method (21). Briefly, the enzyme activities were measured with different concentrations of the substrate (HHL) and the ACE inhibitory pattern in the presence of the inhibitor was determined with a Lineweaver–Burk plot.

### Stability of the Purified Peptide Against *in vitro* Gastrointestinal Digest

*In vitro* digestion of the potent purified ACE inhibitory peptide was carried out according to the reported method, with some adjustments (22, 23). Peptide (0.4 mL, 1.5 mg mL^−1^) was incubated with 2% (w/w) α-amylase, pepsin, or trypsin at 37°C.

In successive digestion tests, the peptide was first dissolved in buffer solution (pH 6.8) to 2 mg mL^−1^ and incubated with α-amylase for 5 min. Then the pH was decreased to pH 2 with the addition of 1 M HCl. Subsequently, the gastric digestion was initiated by adding pepsin at an enzyme-substrate ratio of 2:100 (w/w). The reaction was stopped after 2 h by raising the pH to 8.0 with 1 M NaOH, and trypsin was added to the reactor to continue gastrointestinal digestion for 4 h. Last, the reaction mixture was heated for 15 min in boiling water to inactivate the enzyme.

### Purified Peptide Modulates Angiotensin II Effects on Vascular Smooth Muscle Cells

#### Cell Culture

The rat aortic vascular smooth muscle cell line A7r5, was purchased from Cell bank of Chinese Academy of Sciences (Shanghai, China). A7r5 cells between passage 8 and 20 were grown in DMEM supplemented with 10% FBS and antibiotics until they reached confluence. For experiments, the confluent cells were placed in a quiescing medium (DMEM + 1% FBS + antibiotics) (24) for 24 h, before treatment with different concentrations of FPQYLQYPY and Ang II for different time periods.

#### Cell Cytotoxicity Test

The cytotoxic effects of FPQYLQYPY in cell culture were evaluated using the Cell Counting Kit-8. First, A7r5 cells were seeded at 1 × 104 cells per well into 96-well plates (Costar, Corning, NY, USA) and cultured in a 5% CO_2_ incubator at 37°C for 24 h. Then, the medium of each well was replaced by 100 μL of fresh medium with different concentrations of FPQYLQYPY (0, 1, 5, 10, 50, 100, or 200 μg mL^−1^). After further incubation for 24 h, 10 μL CCK-8 solution was added to each well. Then, the plate was incubated for another 2 h. The absorbance of samples at a wavelength of 450 nm was measured using a microplate reader. The cell survival rate (%) was determined by the percentage of living cells in the test wells compared to the control wells.

#### Cell Proliferation Assay

Cell proliferation was measured by the Cell Counting Kit-8. A7r5 cells were seeded at 5 × 10^3^ cells per well into 96-well plates and grown in DMEM with 10% of FBS for 24 h, then the medium was replaced by quiescing medium overnight. The cells were co-treated with different concentrations of FPQYLQYPY (0, 10, 50, 100 μg mL^−1^) and Ang II (0.1 μM) for 24 h. The absorbance of samples at a wavelength of 450 nm was measured using a microplate reader. The cell survival rate (%) was determined by the percentage of living cells in the test wells compared to the control wells.

#### Wound Healing Assay

Migration of A7r5 cells was evaluated using the wound healing assay (25). Three lines were drawn horizontally on the back of each hole on the 6-well plate with a marker. Cells in logarithmic growth phase were evenly seeded into 6-well plates with 2 × 10^5^ cells per well, and grown in complete medium until the cells covered the bottom of the plate, then the medium was replaced by quiescing medium for overnight.

Afterwards, a sterile 200 μL spear head was used to quickly delimit at an average equal distance in the vertical direction according to the marking line, and washed with PBS to remove the floating cells. Quiescing medium with FPQYLQYPY (0, 10, 50, 100 μg mL^−1^) and Ang II (1 μM) was added, then the cells were cultured in an incubator at 37°C and 5% CO_2_ for 24 h. The degree of cell migration was observed with inverted microscope and photographs were taken at 0 and 24 h. The migration distance was analyzed by ImageJ software.

#### Transwell Cell Migration Assay

A7r5 cells were collected and suspended in DMEM (1% FBS), and then the density was adjusted to 2 × 10^5^ cells ml^−1^. In each group, cell suspensions with or without FPQYLQYPY were cultured in the upper chamber of a Transwell (8.0 μm, Corning, NY, USA) (26), and at the same time, the lower chamber was filled with or without Ang II for 48 h. The superior layer cells of the upper chamber were washed with PBS for 3 times, the cells were then fixed with 4% paraformaldehyde, and stained with 0.1% crystal violet. After rinsing with PBS, a cotton swab was used to remove the upper unmigrated cells. The migratory cells were observed and photographed under an inverted microscope. Last, a 33% acetic acid solution was added to quantify the crystal violet, and the absorbance of samples was measured at a wavelength of 570 nm using a microplate reader.

### RNA Extraction and RNA-Seq Transcriptomics

A7r5 cells were incubated with FPQYLQYPY and Ang II for 24 h. Then, total RNA was extracted using the TRIzol reagent (Thermo Scientific, USA), according to the manufacturer's protocol. After the quality test, the libraries were constructed using the TruSeq Stranded mRNA LT Sample Prep Kit (Illumina, San Diego, CA, USA) following the manufacturer's instructions. The transcriptome sequencing and analysis were conducted by OE Biotech Co., Ltd. (Shanghai, China).

### Real Time Quantitative RT-PCR

Total RNA was extracted from A7r5 cells using TRIzol reagent, according to the manufacturer's specifications. The information of primer sequences is shown in Supplementary Table 1. RT reactions were performed in a GeneAmp^®^ PCR System 9700 (Applied Biosystems, USA). Then, Real-time PCR was performed using the LightCycler^®^ 480 II Real-time PCR Instrument (Roche, Swiss). The relative expression level was calculated using the 2^−Δ*ΔCt*^.

### Statistics

All data are expressed as means ± standard deviation (SD). Statistical significance among multiple experimental groups was analyzed by ANOVA coupled with Tukey's *post-hoc* test using SPSS (version 22). *P* < 0.05 were considered statistically significant.

## Results

### Separation and Purification of ACE-Inhibitory Peptide

**Figure 1:**
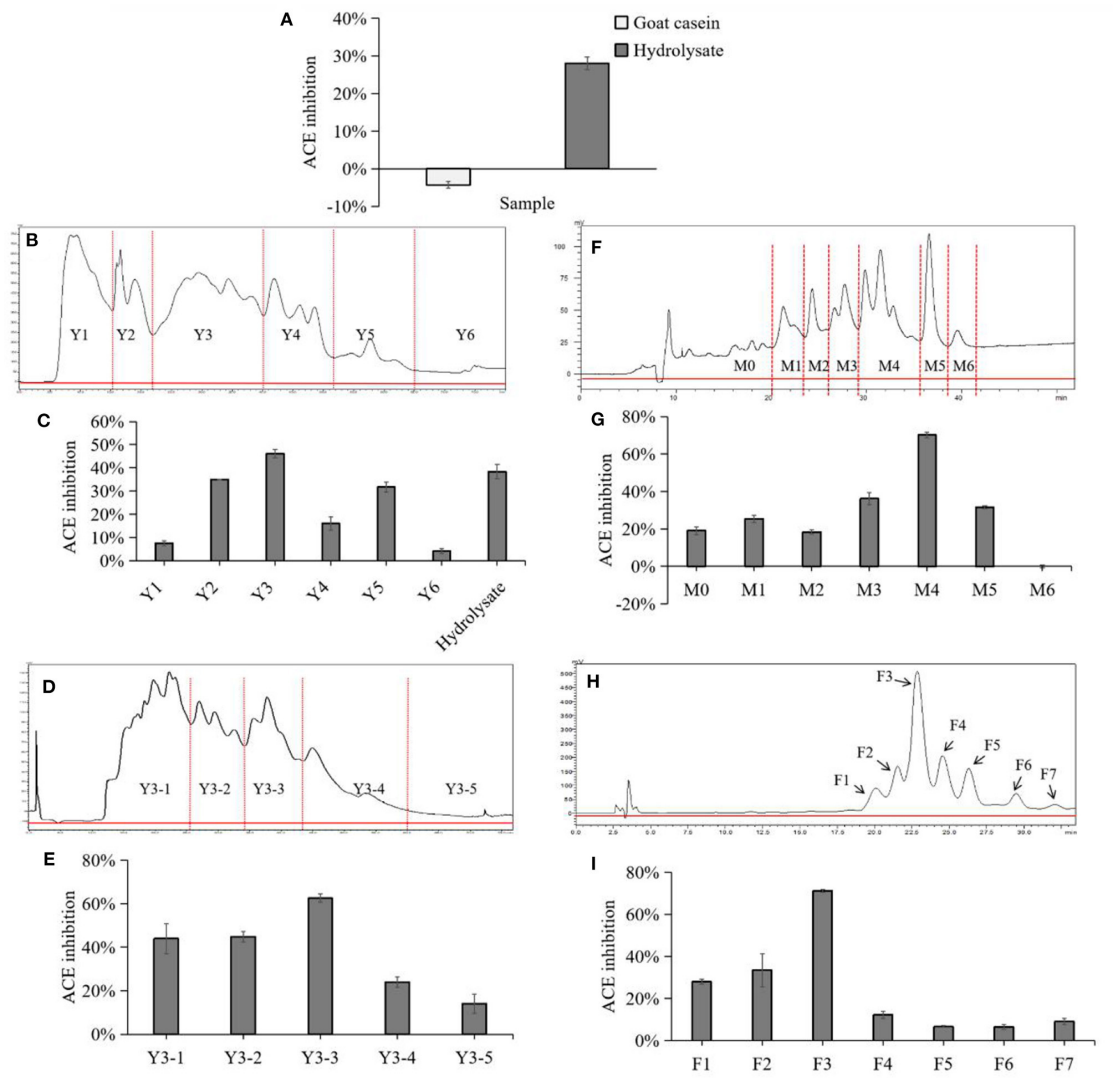
Chromatogram and ACE inhibitory activity of each fraction in separation and purification. **(A)** Comparison of ACE-inhibitory activity before and after casein hydrolysis. **(B)** Preparative HPLC chromatograph of goat milk casein hydrolysates in the first fractionation. Fractions were termed with Y1 to Y6 followed by a number. **(C)** ACE-inhibitory ratios of the collected fractions after the first fractionation and hydrolysate. **(D)** Preparative HPLC chromatograph of goat milk casein hydrolysates in the second fractionation. **(E)** ACE-inhibitory ratios of the collected fractions by the second fractionation. Fractions were termed with Y3-1 to Y3-5 followed by a number. **(F)** Preparative HPLC chromatograph of goat milk casein hydrolysates in the third fractionation. **(G)** ACE-inhibitory ratios of the collected fractions by the third fractionation. Fractions were termed with M0 to M6 followed by a number. **(H)** Separation of the active fraction M4 by analytical RP-HPLC. **(I)** ACE-inhibitory ratios of the collected peaks, and peaks were numbered sequentially from F1 to F7.

Before separating the enzymatic hydrolysate, the ACE-inhibitory activity of goat milk casein and its enzymatic hydrolysate was first compared. It was found that the casein had no ACE-inhibitory ability, but the product after trypsin enzymolysis of casein had certain activity (Figure 1A), indicating that the effective fragments in the protein were released by hydrolysis. RP-HPLC is commonly used to isolate a bioactive peptide (27–29). It has been successfully applied in the separation of ACE-inhibitory peptides from whey protein hydrolysate and casein hydrolysates. Still, the composition of goat casein hydrolysate is complicated. In order to filtrate out the highest active peptides, this study adopted a four-step separation method to purify the monomer peptides with the best ACE-inhibitory activity.

**Figure 2:**
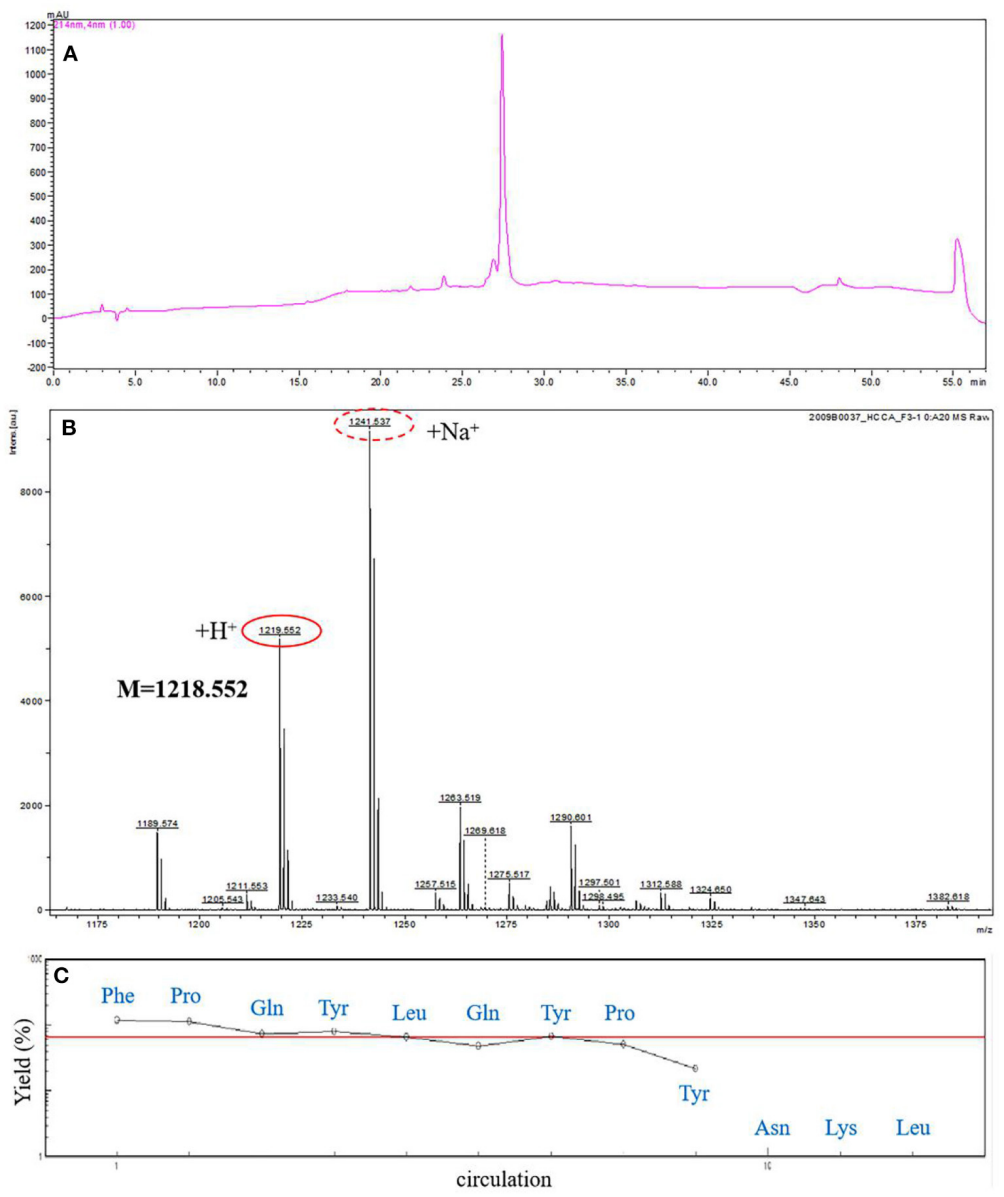
Amino acid sequence analysis of F3. **(A)** Further analysis of F3 by analytical RP-HPLC. **(B)** The molecular mass analysis of the ACE-inhibitory peptide F3 by MALDI-TOF-MS/MS. **(C)** The N-terminal sequence analysis of F3 by PPSQ.

In this study, the ACE-inhibitory activity of the hydrolysate was determined before the separation process. The hydrolysate was divided into six fractions by preparative reversed-phase C18 column, and fraction Y3 showed a highest inhibition which was notably higher than the hydrolysate (Figures 1B,C). Then, Y3 was further separated with a RP Shim-pack PRC-ODS(K) column and divided into 5 fractions, and Y3-3 had the highest inhibition rate of 63.58 ± 1.91% (Figures 1D,E). Next, Y3-3 was divided into six fractions further purified by a RP ECOSIL C18 column (Figures 1F,G). Finally, in order to improve the level of purity in the M4 fraction with the highest inhibition in the previous step, RP-HPLC equipped with a C-18 column was used to purify M4 with a gradient elution. Seven peaks were obtained based on differences in polarity, F3 exhibited the best ACE-inhibitory ability (71.19 ± 0.71%) (Figures 1H,I) and was regarded as a monomer. F3 was further analyzed by analytical HPLC with the same C18 column, and it was shown that the fraction was single peak with high purity (Figure 2A).

### Identification of the ACE Inhibitory Peptide

In order to obtain the exact sequence of the purified peptide, MALDI-TOF-MS/MS combined with Protein Sequencer (PPSQ) was used. According to MALDI-TOF-MS/MS analysis, the molecular mass of ACE inhibitory peptide F3 was 1218.552 Da (Figure 2B). As shown in Figure 2C, the N-terminal amino acid sequence of F3 was analyzed by Protein Sequencer, and peptide F3 was composed of nine amino acids with the sequence identified as Phe-Pro-Gln-Tyr-Leu-Gln-Tyr-Pro-Tyr (FPQYLQYPY). The peptide of this sequence was derived from α-casein, as determined by a search of the protein database of UniProt. The results were consistent with those reported previously that most ACE inhibitory peptides have amino acid residues between 2 and 12 (30) and molecular weight below 3 kDa (31).

### Inhibition Pattern of FPQYLQYPY

**Figure 3:**
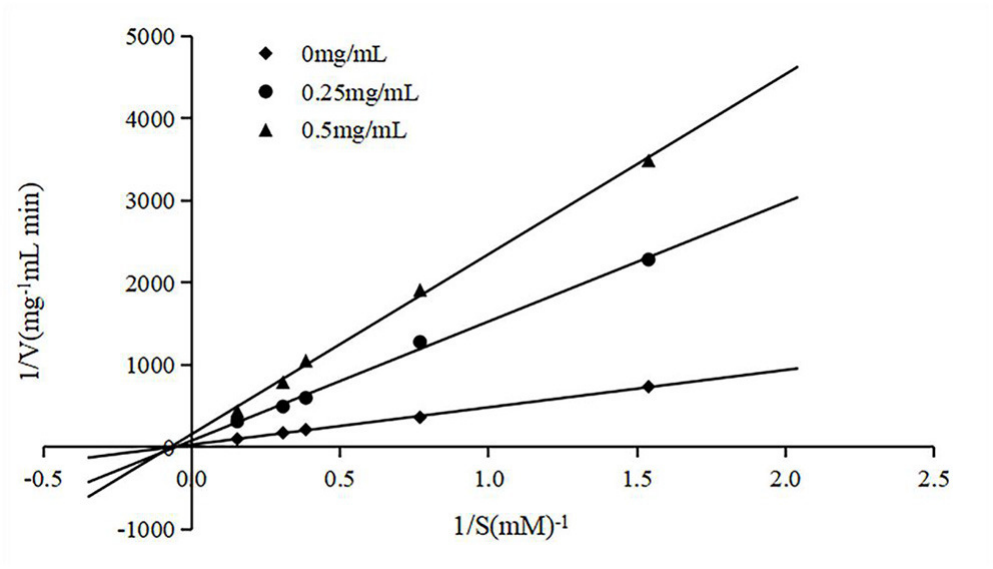
Lineweaver-Burk plots of ACE inhibition by F3.

To clarify the type of inhibition, the peptide was co-incubated with various substrate (HHL) concentrations and an ACE solution, and the double reciprocal velocity-substrate is shown in Figure 3. The Michaelis-Menten equation can reflect the relationship between the initial velocity and the substrate concentration in an enzymatic reaction. Usually, the K_m_ and V_max_ for the reaction at different concentrations of the peptide were determined according to Lineweaver–Burk plots. As Km and Vm are important parameters of enzymatic reactions, the inhibition types of ACE-inhibitory peptides can be determined by them. There are two common inhibition types of ACE-inhibitory peptides, competitive inhibition and non-competitive inhibition. As shown in Figure 3, the lines intersected at similar x-intercepts, so the inhibition method of the peptide to ACE was determined to be non-competitive, suggesting that the peptide could bind to the enzyme at a site other than the active site without competition between the substrate and the enzyme.

### Stability of the FPQYLQYPY Against *in vitro* Gastrointestinal Digest

The stability of the peptide against gastrointestinal digest was assessed *in vitro*. FPQYLQYPY was progressively incubated with various digestive enzymes, including α-amylase, pepsin, and trypsin. Then, the ACE inhibitory activity was assessed, and we observed that peptide digestion by α-amylase and pepsin promoted ACE inhibitory activity slightly when compared with the original (with inactivation of enzymes at every stage). However, peptide digestion by trypsin after 4 h reduced the ACE inhibitory activity a bit (Table 1). Despite these trends, there was no significant difference in activity after incubation with the digestive enzyme. These results suggested that the bioactivity of FPQYLQYPY may be resistant to digestion in the gastrointestinal tract.

**Table 1:** ACE inhibitory activity of FPQYLQYPY after *in vitro* gastrointestinal digest.

**Digestion step**	**Sample**	**ACE inhibitory (%)**
Oral digestion (α-Amylase)	Original	53.47 ± 7.32
	Digested	57.95 ± 0.22
Gastric digestion (Pepsin)	Original	54.12 ± 1.56
	Digested	56.41 ± 1.91
Intestinal digestion (Trypsin)	Original	49.86 ± 1.68
	Digested	45.76 ± 0.42

### Cytotoxicity Test of FPQYLQYPY in VSMCs

**Figure 4:**
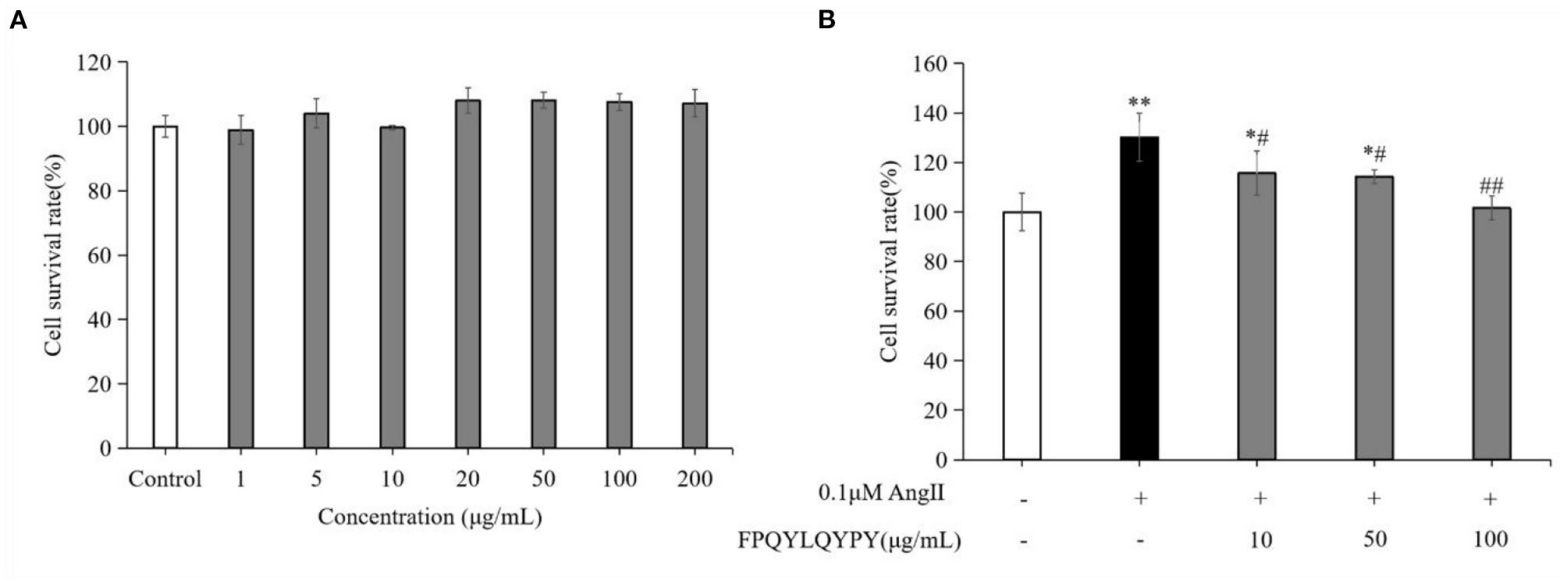
FPQYLQYPY inhibited proliferation of Ang II-stimulated VSMCs. **(A)** Cytotoxicity of FPQYLQYPY in VSMCs cells measured by CCK-8. **(B)** FPQYLQYPY inhibited Ang II-stimulated proliferation of VSMCs. Data are expressed as mean ± SD. *P < 0.05 and **P < 0.01, respectively, as compared to the control group. ^#^*P* < 0.05 and ^*##*^*P* < 0.01, respectively, as compared to the Ang II-treated group.

The cytotoxicity test result (Figure 4A) showed that FPQYLQYPY in the concentration range of 1–200 μg mL^−1^ exhibited no obvious cytotoxic effect on A7r5 cells (*P* > 0.05). And this test results provided a reference for the selection of the peptide concentration in the subsequent experiments.

### FPQYLQYPY Inhibited Ang II-Stimulated Proliferation of VSMCs

The proliferation of cultured A7r5 cells was determined by CCK-8 assay. As expected, stimulation with Ang II for 24 h significantly increased the proliferation of VSMCs (Figure 4B). Treatment with FPQYLQYPY significantly decreased the percentage of proliferating cells, indicating the potential ability of this peptide for inhibiting excessive proliferation of Ang II-stimulated VSMCs.

### The Inhibition of Ang II-Stimulated Migration of VSMCs by FPQYLQYPY

**Figure 5:**
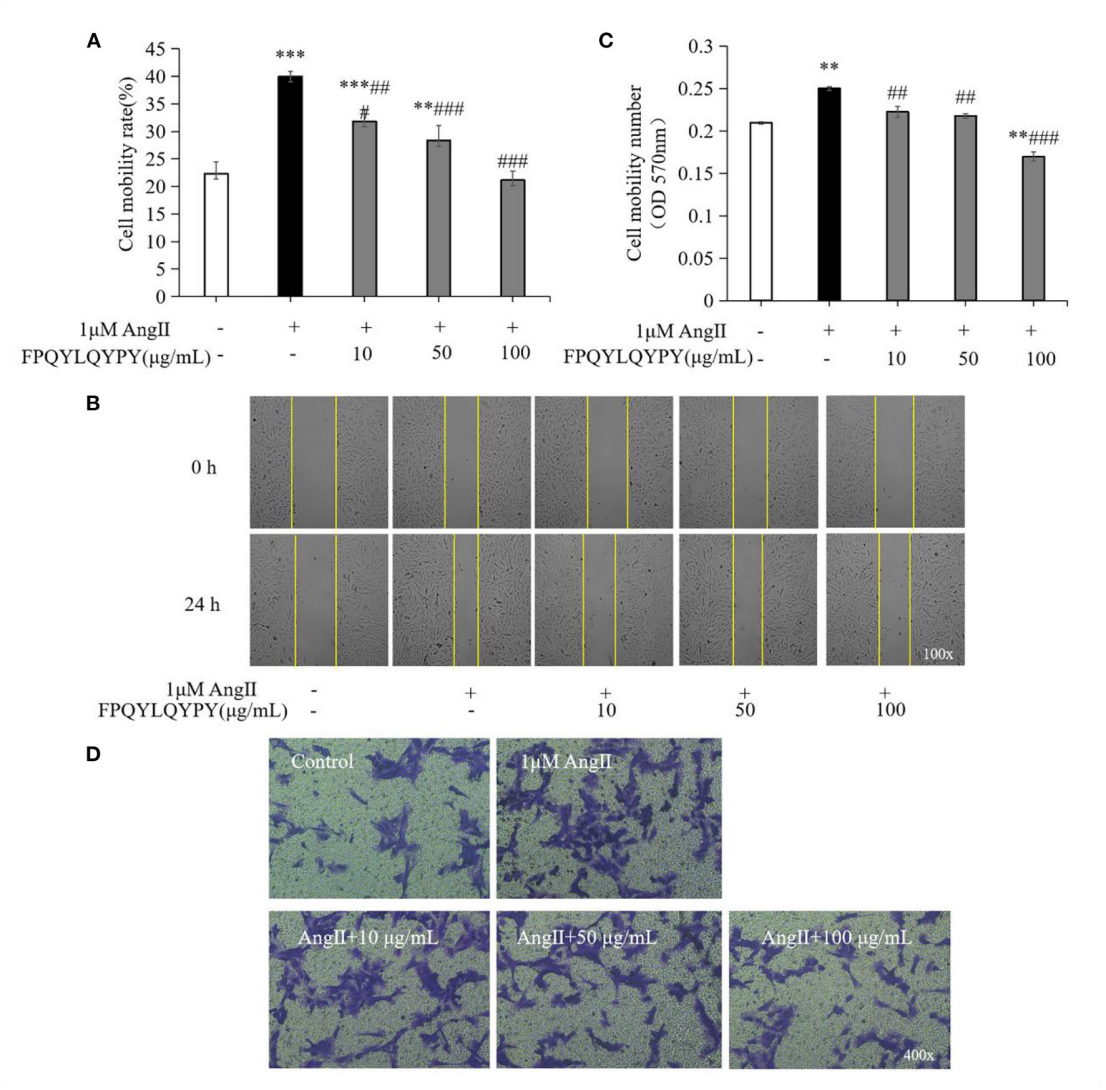
FPQYLQYPY inhibited migration of Ang II-stimulated VSMCs. **(A,B)** Cell migration was evaluated by wound healing assay, and the distance of the wound healing area was measured by ImageJ software. **(C,D)** Cell migration was evaluated by Transwell experiments. All the data are represented as the mean ± SD. **P < 0.01 and ***P < 0.001, respectively, as compared to the control group. ^*##*^*P* < 0.01 and ^*###*^*P* < 0.001, respectively, as compared to the Ang II-treated group.

The migration of VSMCs was measured by wound healing assay and Transwell assay. Stimulation of Ang II could promote the migration of A7r5 cells (Figure 5), while co-treatment with FPQYLQYPY could adjust the migration ability of Ang II-stimulated A7r5 cells. FPQYLQYPY at a concentration of 100 μg mL^−1^ could restore the distance of the wound healing area to the level of the control group (Figures 5A,B).

Furthermore, Transwell experiments further revealed that Ang II caused a significant increase in the number of cells passing through the chamber (*P* < 0.01). However, after 48 h treatment with FPQYLQYPY, the decrease in the level of cell migration was noteworthy (Figures 5C,D). These two experiments show that FPQYLQYPY can adjust the migration of Ang II -stimulated VSMCs.

### Transcriptomics Responses of FPQYLQYPY to Ang II-Stimulated VSMCs

**Figure 6:**
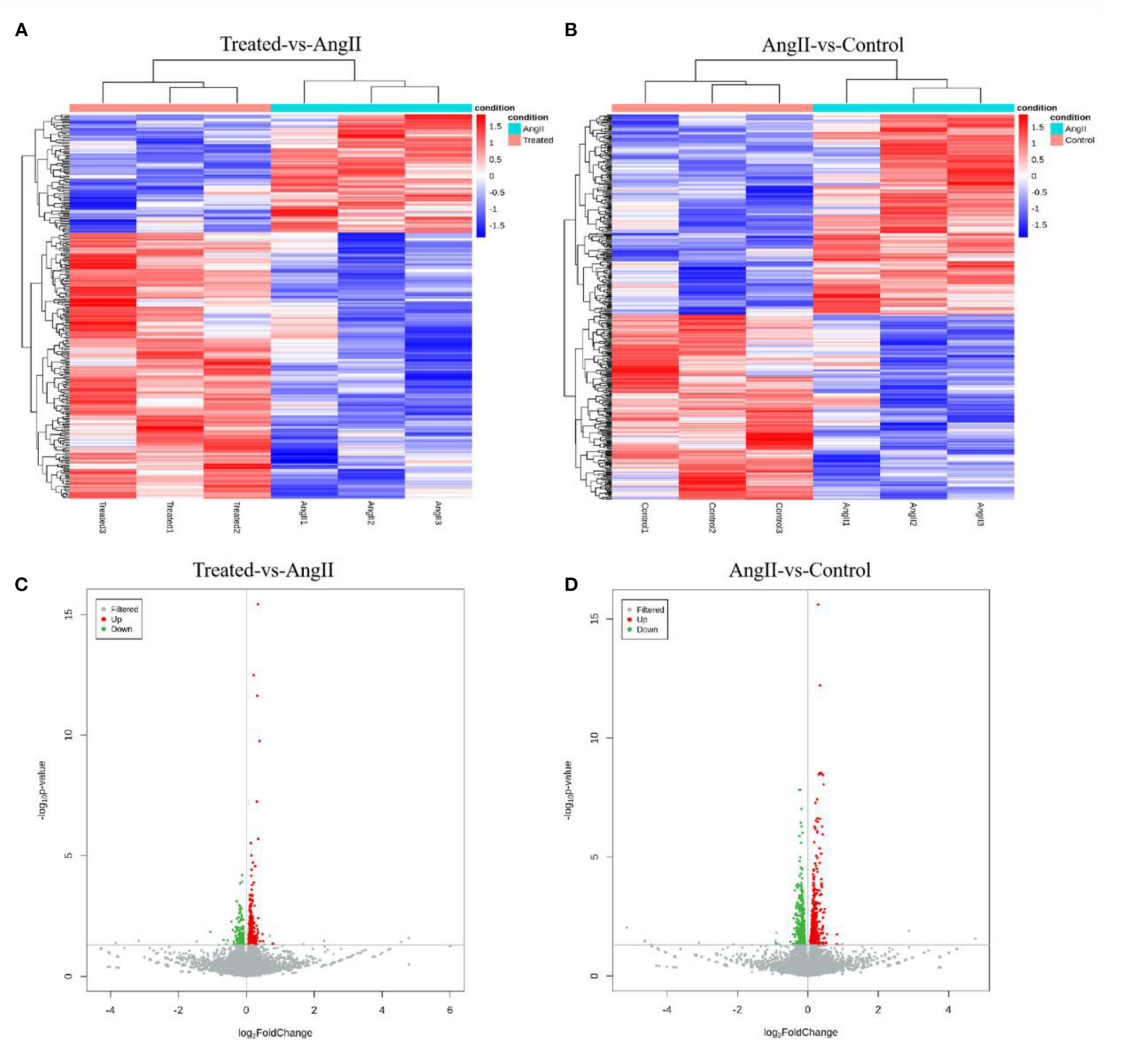
Transcriptome gene expression altered in the VSMCs. **(A)** Hierarchal clustering of differentially expressed genes (DEGs) in FPQYLQYPY treated group and Ang II-treated group. **(B)** Hierarchal clustering of differentially expressed genes (DEGs) in Ang II-treated group and control group. **(C)** The volcano map of differentially expressed genes (DEGs) in FPQYLQYPY treated group and Ang II-treated group. **(D)** The volcano map of differentially expressed genes (DEGs) in Ang II-treated group and control group.

Transcriptome sequencing was performed using RNA from A7r5 cells. The typically changed genes were identified and shown in the heatmaps and volcano maps (Figure 6). The results showed that 342 differentially expressed genes (DEGs) (*P* < 0.05) were detected between the FPQYLQYPY treated group and the Ang II-treated group, and 797 genes between the Ang II-treated group and the control group.

**Figure 7:**
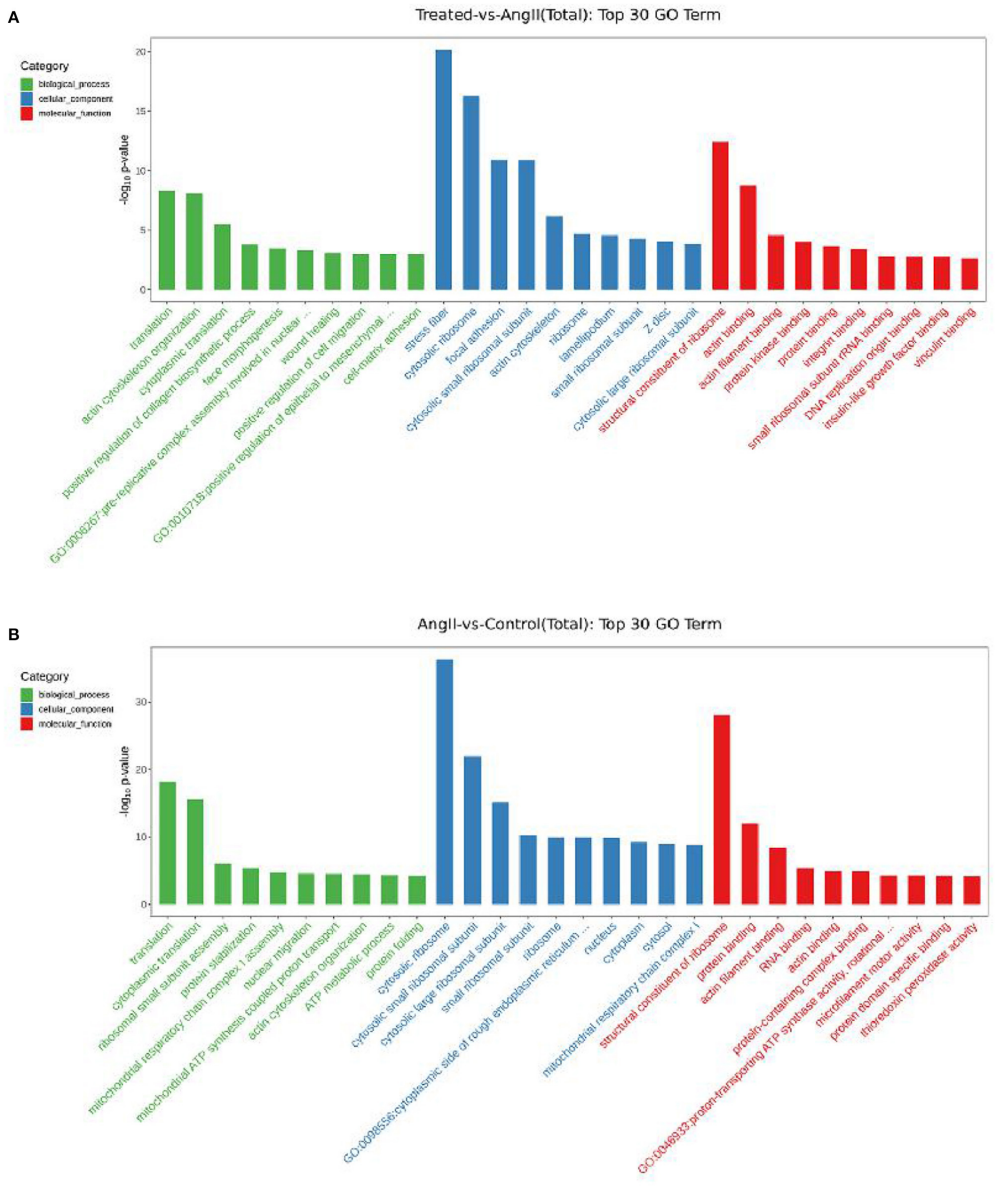
Gene Ontology (GO) enrichment analysis for differentially expressed genes (DEGs). **(A)** Top 30 GO-terms of DEGs in FPQYLQYPY treated group and Ang II-treated group. **(B)** Top 30 GO-terms of DEGs in the Ang II-treated group and control group.

After obtaining the differentially expressed genes, GO enrichment analysis was performed in order to describe their functions, covered biological processes, cellular components, and molecular functions. There were 212 significantly upregulated and 117 significantly downregulated genes in the comparison of the FPQYLQYPY and Ang II-treated groups. The top 30 GO-terms of significantly differential genes were mainly involved in stress fiber, actin cytoskeleton organization, positive regulation of cell migration, wound healing, and actin binding. These enriched GO terms may be related to vascular remodeling. Furthermore, the top 30 GO-terms of DEGs in the Ang II-treated group and the control group are shown in Figure 7. The DEGs were mainly involved in cytosolic ribosome, cytoplasmic translation, structural constituent of ribosome, and actin filament binding.

**Figure 8:**
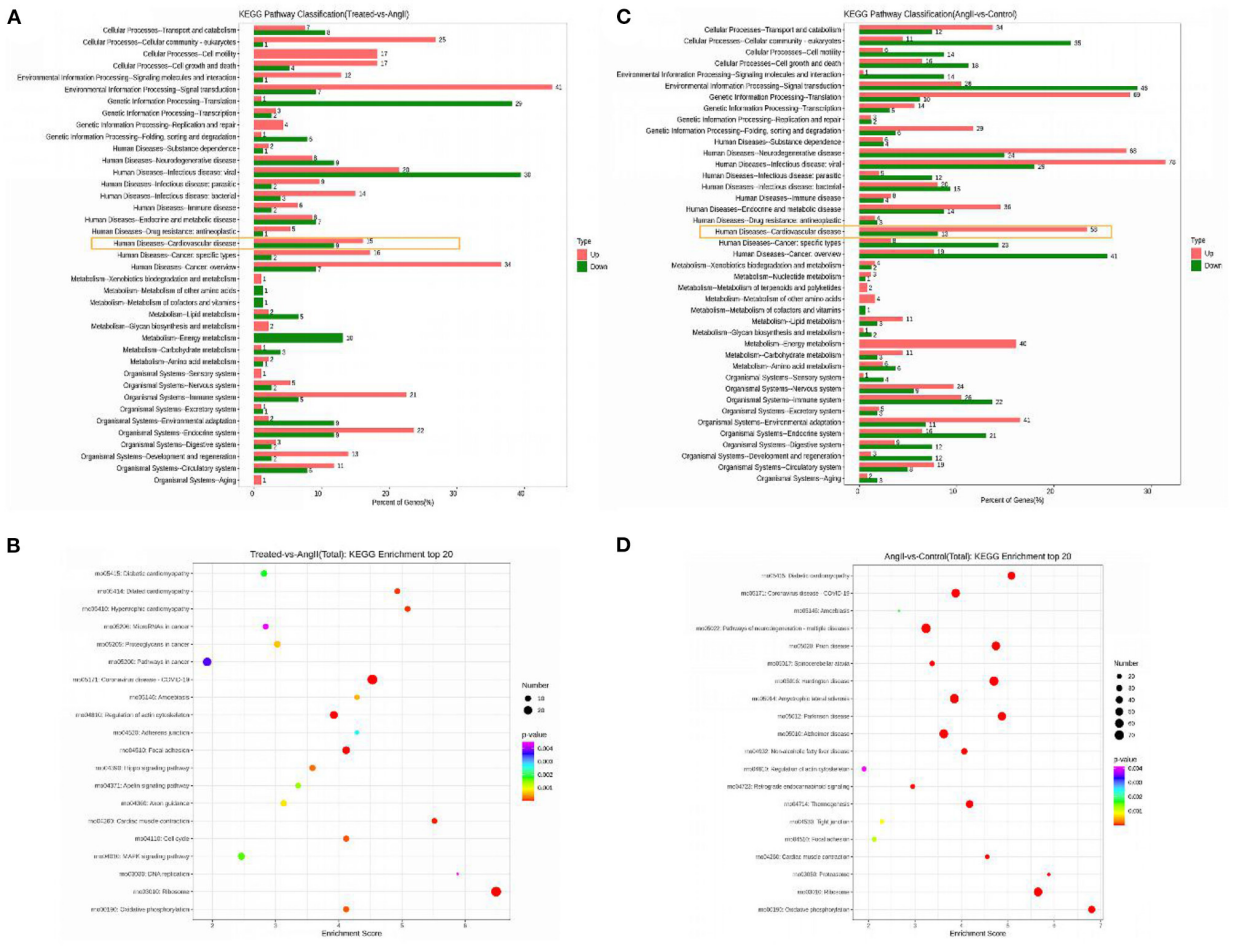
Kyoto Encyclopedia of Genes and Genomes (KEGG) enrichment analysis for differentially expressed genes (DEGs). **(A)** KEGG pathway classification of DEGs in the FPQYLQYPY treated group and Ang II-treated group. **(B)** Bubble chart of the top 20 significantly enriched pathways for DEGs in the FPQYLQYPY treated group and Ang II-treated group. **(C)** KEGG pathway classification of DEGs in the Ang II-treated group and control group. **(D)** Bubble chart of the top 20 significantly enriched pathways for DEGs in the Ang II-treated group and control group.

Pathway analysis using KEGG can further appreciate the function of genes and their interactions. Differential genes were enriched in cardiovascular disease-related pathways between the FPQYLQYPY-treated group and the Ang II-treated group, and between the Ang II-treated group and the control group. The top 20 enriched pathways in the FPQYLQYPY-treated group and the Ang II-treated group are presented in Figure 8 and were involved in diabetic cardiomyopathy, dilated cardiomyopathy, hypertrophic cardiomyopathy, regulation of actin cytoskeleton, cardiac muscle contraction, MAPK signaling pathway, and vascular smooth muscle contraction. However, the KEGG pathway enrichment analysis of the DEGs in the Ang II-treated group and the control group revealed diabetic cardiomyopathy, pathways of neurodegeneration, multiple diseases, cardiac muscle contraction, and the ribosome.

Overall, the analysis of pathways showed that the ACE inhibitory peptide may have a regulatory effect on Ang II -induced changes in cardiovascular-related pathways at the cellular level.

### Verification of Differentially Expressed Genes by RT-PCR

**Figure 9:**
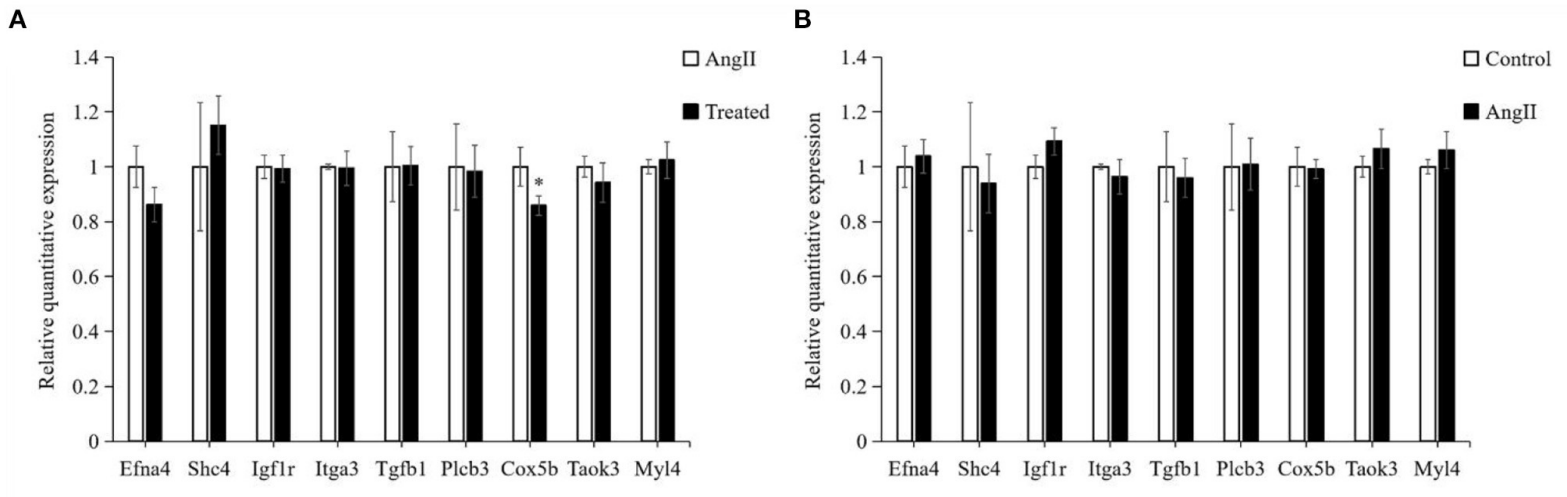
Verification of identified differentially expressed genes (DEGs) in VSMCs by RT-PCR. **(A)** mRNA expression of identified DEGs in the FPQYLQYPY treated group and Ang II-treated group. **(B)** mRNA expression of identified DEGs in the Ang II-treated group and control group. All the data are represented as the mean ± SD. *P < 0.05.

Based on the GO and pathway results, we selected 9 genes among all identified DEGs for verification by RT-PCR. As shown in Figure 9. Efna4, Igf1r, Itga3, Plcb3, Cox5b, and Taok3 were downregulated, whereas Shc4, Tgfb1, and Myl4 were upregulated in the comparison of FPQYLQYPY-treated group and the Ang II-treated group. Although, in the comparison of the Ang II-treated group and the control group, Shc4, Itga3, Tgfb1, and Cox5b were downregulated, but Efna4, Igf1r, Plcb3, Taok3, and Myl4 were upregulated. These results are consistent with transcriptome sequencing.

## Discussion

Enzyme hydrolysis is a commonly used method to obtain biologically active peptides from milk proteins (32). Compared with unhydrolyzed proteins, these soluble hydrolysates exhibited stronger ACE inhibition. In the present work, ACE inhibitory peptides were produced *in vitro* using gastrointestinal enzymes (usually pepsin, trypsin, or α-chymotrypsin). In previous studies, the effective ACE inhibitory peptides released from goat whey protein and casein by pepsin hydrolysis, PEQSLACQCL of β-lactoglobulin (f 113-122), QSLVYPFTGPI of β-casein (residue 56-66), and ARHPHLSFM of κ-casein (f 96-106), were first introduced by Ibrahim et al. (33). In this study, we used trypsin to hydrolyze goat milk caseins to release the peptides with ACE-inhibitory activity, and used RP-HPLC to gradually separate hydrolysates based on difference in polarity. The ACE inhibitory peptide F3 showed the strongest ACE inhibitory activity and was obtained after separation for four times (Figure 1I).

The *in vivo* antihypertensive effects of most ACE-inhibitory peptides have been attributed to their low molecular weight, and short amino acid sequences. For example, after fermenting goat milk with *Lactobacillus casei*, the supernatant was ultrafiltered with 3, 5, and 10 kDa ultrafiltration membranes to obtain different permeate and retentate samples, of which the 3 kDa sample showed the highest ACE-inhibitory activity (34). Aslam et al. (35) used ultrafiltration to separate five peptides with a higher concentration and a molecular weight <3 kDa in fermented goat milk. Among them, VLPVPQKAVPQ and VLPVPQKVVPQ had good ACE-inhibitory activity, and they were composed of <12 amino acids (35). Furthermore, the ACE-inhibitory activity of peptides seemed to rely on their amino acid composition and hydrophobicity. The somatic-ACE consisted of two homologous domains, N- and C-domains (36), which seemed to play an important role in blood pressure regulation (37). Most of the ACE-binding peptides were strongly influenced by the residues in the three positions closest to the C-terminal. In particular, the peptides with hydrophobic amino acids at the C-terminal showed prominent ACE-inhibitory activity, including Phe, Trp, Tyr, Val, Leu, Ile, and Pro, were more favorable for binding with ACE. Besides, amino acid sequences with higher Pro content could enhance the ACE inhibition effect and as well as intensify the strong digestive resistance of the gastrointestinal tract (7). VPP and IPP conformed to these characteristics, and they are the most widely studied ACE inhibitory peptides. They are highly resistant to digestion in the gastrointestinal tract, can reach the small intestine in complete form (38), and have been shown to improve blood pressure in hypertensive patients (39).

In this study, we identified that the peptide with the highest ACE inhibitory activity was Phe-Pro-Gln-Tyr-Leu-Gln-Tyr-Pro-Tyr (FPQYLQYPY), which was derived from α-casein of goat milk (Figure 2). Based on currently available information, this peptide has not been previously reported. This peptide had a low molecular weight with many hydrophobic residues, was rich in proline, and ended with the hydrophobic amino acid Tyr at its C-terminal. Furthermore, it was consistent with the characteristics of high ACE-inhibitory activity sequence as described above, and this may indicate a structure-activity relationship, although more research is needed.

*In vitro* gastrointestinal enzyme simulation can simply describe the changes of a peptide during oral administration (40). After the digestion, we found that the hydrolysate still had ACE inhibitory activity (Table 1), however, we were still unclear whether the peptide was hydrolyzed to the active fragment, or changed to the fragment in this digestive process. These topics will be the focus of future study. We also investigated the inhibition pattern of FPQYLQYPY and confirmed the inhibitory model of the peptide as non-competitive (Figure 3), which was found to be the common inhibition pattern of ACE inhibitory peptides in previous reports (41, 42).

Due to the important role of ACE in hydrolyzing angiotensin I into angiotensin II under the condition of strong vasoconstriction, ACE-inhibition has always been a key target of anti-hypertensive research (43, 44). It has been stated that ACE-inhibitory peptides have a variety of blood pressure-lowering effects other than ACE inhibition (8). Previous reports indicate that the effect of angiotensin II on VSMCs is widely used to explore the mechanism of cardiovascular disease (45). Therefore, in this study, the anti-hypertension effects of the purified peptide *in vitro* were investigated by studying its modulation of the effects on angiotensin II-stimulated vascular smooth muscle cells.

Dysfunction of VSMCs can be induced by Ang II and results in adverse events like increased oxidative stress, inflammation, migration, and hyperplasia, and therefore plays a key role in the pathogenesis of hypertension, restenosis, and atherosclerosis (46, 47). The pathological proliferation and migration of VSMCs induced by Ang II are the dominative factor of vascular remodeling (43). Studies have found that antihypertensive peptides derived from egg white can inhibit migration and regulate Ang II-stimulated VSMCs (48, 49). Aberrant proliferation and migration of vascular smooth muscle cells are a major pathological phenomenon in hypertension (45). In this study, we found that co-incubation with FPQYLQYPY could significantly inhibition the excessive proliferation and migration of Ang II-stimulated VSMCs (Figures 4B, 5). These result indicated that FPQYLQYPY or other ACE inhibitory peptides may improve the vascular remodeling induced by Ang II.

Transcriptome sequencing analysis revealed the mechanism of the peptide effects on Ang II-stimulated VSMCs, the differentially expressed genes were analysis by GO and KEGG enrichment to determine their mainly biological functions or pathways. The results showed that, the differentially expressed genes were enriched in cardiovascular disease-related pathways, mainly involved in cardiomyopathy, cardiac muscle contraction, and vascular smooth muscle contraction. The differentially expressed genes, such as Itga3 of the integrin family and transforming growth factor beta-1 (Tgfb-1) and insulin-like growth factor 1 receptor (Igf-1R) of the growth factor family, and these genes tend to affect cell proliferation and migration, they are likely to cause fibrotic effects on vascular tissue or angiogenesis (50, 51). At the same time, the peptide also regulated the expression of the Efna4 and Taok3 genes in the MAPK signaling pathway, which has important functions in cardiovascular disease, can repair vascular injury, and is involved in cardiac remodeling (52). Moreover, Pcb3, Cox5b, and Myl4 are involved in atherosclerosis, diabetic cardiomyopathy, and cardiac muscle contraction, respectively. Furthermore, changes in insulin signaling mediated by Shc4 may be involved in the process of cardiac development (53, 54).

The pathways in which these DEGs are involved suggest that the peptide had the ability to modulate cellular dysfunction caused by Ang II on VSMCs. And the regulatory effects of this peptide were manifested in vascular remodeling, cardiac remodeling, and cardiomyopathy. These results correspond to the inhibitory ability of the peptide on excessive proliferation and migration of the Ang II-stimulated VSMCs shown in experiments. Simultaneously, our findings illustrated other antihypertensive mechanisms of this peptide *in vitro*.

## Conclusion

In conclusion, a novel ACE-inhibitory peptide was separated and purified from goat milk casein hydrolysates, and the amino acid sequence was identified as FPQYLQYPY. The bioactivity of the hydrolysate persisted against *in vitro* gastrointestinal digest, and the ACE-inhibitory model of the peptide was confirmed as non-competitive. The anti-hypertension of FPQYLQYPY was evaluated by Ang II-stimulated VSMCs *in vitro*. This peptide could inhibit the excessive proliferation and migration of VSMCs. Besides, transcriptome sequencing analysis revealed that DEGs were enriched in cardiovascular disease-related pathways and implicated the regulatory effects of the peptide on vascular remodeling. The results of this study show that, in addition to inhibiting ACE, this peptide also regulates vascular dysfunction, which may broaden the application of the ACE-inhibitory peptides as functional food ingredients. However, the precise antihypertensive mechanism of this peptide remain unclear, and more studies are needed in the future to fully understand this process.

## Data Availability Statement

The datasets presented in this study can be found in online repositories. The names of the repository/repositories and accession number(s) can be found below: NCBI; PRJNA809493

## Author Contributions

ZQ: data curation, formal analysis, investigation, writing—review and editing, and validation. JW, LP, XP, YG, and SC: funding acquisition, resources, and validation. ZH and QL: data curation, formal analysis, and investigation. KF: investigation and writing—review and editing. MS: methodology, writing—reviewing and editing, and investigation. YCh: investigation, writing—review and editing, and supervision. YCa: project administration, funding acquisition, writing—reviewing and editing, investigation, supervision, and resources. GL: project administration, funding acquisition, writing—reviewing and editing, investigation, and supervision. All authors contributed to the article and approved the submitted version.

## Funding

The authors express their gratitude to the Guangdong Province Engineering Research Center for Bioactive Natural Products (2016B090920093), Guangdong Provincial Key Laboratory of Nutraceuticals and Functional Foods (2018B030322010), the Program for Guangdong Introducing Innovative and Entrepreneurial Teams (2019ZT08N291), the Project funded by China Postdoctoral Science Foundation (2020M672651), and the Innovation Platform and Talent Plan of Hunan Engineering and Technology Research Center for Nutrition and Health Products (2019TP2066).

## Conflict of Interest

JW, LP, XP, YG, and SC were employed by Ausnutria Dairy (China) Co., Ltd. The remaining authors declare that the research was conducted in the absence of any commercial or financial relationships that could be construed as a potential conflict of interest.

## Publisher's Note

All claims expressed in this article are solely those of the authors and do not necessarily represent those of their affiliated organizations, or those of the publisher, the editors and the reviewers. Any product that may be evaluated in this article, or claim that may be made by its manufacturer, is not guaranteed or endorsed by the publisher.
