# Supplementary material for: A Novel Angiotensin I-Converting Enzyme Inhibitory Peptide Derived From Goat Milk Casein Hydrolysate Modulates Angiotensin II-Stimulated Effects on Vascular Smooth Muscle Cells
Source: Front Nutr. 2022 Apr 11;9:878768. doi: 10.3389/fnut.2022.878768 (PMC9037752; doi:10.3389/fnut.2022.878768)
Supplement: Supplementary file 1 [file Table_1.DOCX]

**The information about primer sequences:**

| **Num** | **Gene Symbol** | **Forward primer(5->3)** | **Reverse primer(5->3)** | **Product length(bp)** | **Tm(℃)** |
| --- | --- | --- | --- | --- | --- |
| 1 | Efna4 | GCCACTCTATCTACTGGAACT | TGTCTAGGTAATCGTTGAGGC | 87 | 60 |
| 2 | Shc4 | ACGCTGTATGTAGGACTCT | GACGTGATGGACTCGTTC | 83 | 60 |
| 3 | Igf1r | TGACATCCGCAACGACTA | TGGAGATGAGCAGGATGTG | 86 | 60 |
| 4 | Itga3 | TGGATGATGACTACACCAACC | TCCATGCGTTCACAATCG | 82 | 60 |
| 5 | Tgfb1 | CCGTGGCTTCTAGTGCTGA | CTTCCGTTTCACCAGCTC | 87 | 60 |
| 6 | Plcb3 | AGTCAAGGTCTGGTCAGAG | CTTTCCGAAGGAACGTGT | 86 | 60 |
| 7 | Cox5b | CATTACAAGTTGGTGCCCTAC | GTGCGTTGGCTAGTCTTTAT | 105 | 60 |
| 8 | Taok3 | CCAACGAGGTGGTTGCTATTA | TTCCTTCAGGATATCTTGCCAT | 80 | 60 |
| 9 | Myl4 | GATCCCAAGAGTGTGAAGATAG | GGAGTCCGGTCAAACAAT | 83 | 60 |

**Table 1. The primer sequences**
